# A novel strategy for performing endoscopic submucosal dissection for circumferential superficial esophageal neoplastic lesions with kissing traction

**DOI:** 10.1055/a-2344-7717

**Published:** 2024-07-30

**Authors:** Ben-hua Wu, Li-sheng Wang, Zheng-lei Xu

**Affiliations:** 1 Department of Gastroenterology, Shenzhen People’s Hospital, Shenzhen, China

Endoscopic submucosal dissection (ESD) is a widely utilized procedure for early esophageal cancer and precancerous lesions. However, it remains technically challenging and time consuming for circumferential lesions. Numerous studies have utilized various techniques for improving the efficacy of ESD in the treatment of esophageal circumferential lesions 1 2 3 . Herein, we propose a novel approach for improving ESD for esophageal peripheral lesions, termed the “kissing traction technique” (KT-ESD).

Over 3 years, six patients (four male, two female; mean age 62.5 years) with circumferential superficial esophageal neoplastic lesions underwent KT-ESD. Informed consent for the procedure was signed by all patients before the procedure intervention.

The steps of KT-ESD were as follows ( Video 1 ). 1) Esophageal iodine staining ( Fig. 1 **a** ). 2) Marking the lesion. 3) Marking a circumferential incision on the anal and oral sides of the lesion (0.5 cm away from the lesion). 4) Dissection to reveal the mucosal flap from the oral side of the lesion ( Fig. 1 **b** ). 5) Using two legs of titanium clips to clamp the two kissing mucosal flaps before traction ( Fig. 1 **c** , **d** ), so that only one traction is needed to pull up the whole lesion. 6) Complete dissection along the submucosa. 7) Injection of triamcinolone acetonide solution on the wound; postoperative oral steroids were administered to prevent stenosis.

A novel strategy for performing endoscopic submucosal dissection for circumferential superficial esophageal neoplastic lesions with kissing traction.Video 1

**Fig. 1:**
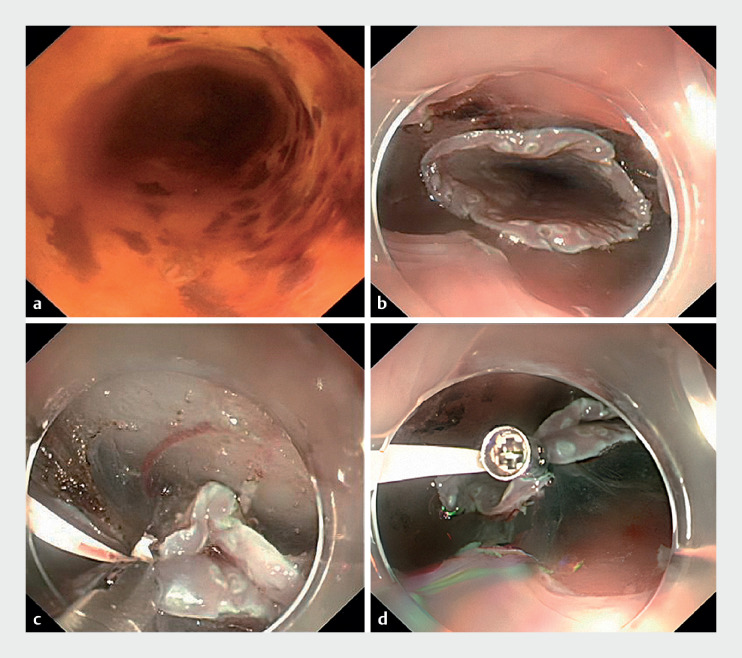
The procedural steps of endoscopic submucosal dissection with kissing traction. **a** Esophageal iodine staining. **b** The mucosal flap from the oral side of the lesion. **c** Using two legs of titanium clips to clamp the two kissing mucosal flaps. **d** Established status of post-kissing traction.

The length of the lesions ranged from 5.0 to 9.5 cm, with operation times ranging from 55 to 92 minutes ( Table 1 ). None of the patients experienced intraoperative or postoperative bleeding or perforation. No recurrence was observed in subsequent follow-up. During the 3–30 months of postoperative follow-up, two of the six patients developed esophageal stenosis; however, the stenosis was successfully relieved following two to three balloon dilation procedures.

**Table:** **Table 1** Characteristics of six patients undergoing endoscopic submucosal dissection with kissing traction.

Sex	Age	Location ^1^	Longitudinal diameter, cm	Macroscopic type	Operative time, minutes	Adverse events	Pathological type	Follow up, months
M	69	Middle and inferior	6	IIb	65	No	Intramucosal cancer	3
F	56	Middle	5	IIb	55	Stenosis	High grade intraepithelial neoplasia	5
F	64	Middle and inferior	6.5	IIb+IIa	70	No	Intramucosal cancer	15
M	56	Middle and inferior	9.5	IIb+IIa	92	Stenosis	Intramucosal cancer	25
M	60	Middle and inferior	7	IIb	80	No	High grade intraepithelial neoplasia	12
M	70	Middle and inferior	5	IIb	60	No	Intramucosal cancer	30
^1^ Superior, middle, and inferior of the esophageal.								

In conclusion, KT-ESD demonstrated a significant improvement in dissection efficiency for peripheral esophageal lesions, without an increase in complications.

Endoscopy_UCTN_Code_TTT_1AO_2AG_3AZ

## Notes

Endoscopy E-Videos https://eref.thieme.de/e-videos
*E-Videos* is an open access online section of the journal *Endoscopy* , reporting on interesting cases and new techniques in gastroenterological endoscopy. All papers include a high-quality video and are published with a Creative Commons CC-BY license. Endoscopy E-Videos qualify for HINARI discounts and waivers and eligibility is automatically checked during the submission process. We grant 100% waivers to articles whose corresponding authors are based in Group A countries and 50% waivers to those who are based in Group B countries as classified by Research4Life (see: https://www.research4life.org/access/eligibility/ ). This section has its own submission website at https://mc.manuscriptcentral.com/e-videos .
